# Mouse IgG2a Isotype Therapeutic Antibodies Elicit Superior Tumor Growth Control Compared with mIgG1 or mIgE

**DOI:** 10.1158/2767-9764.CRC-22-0356

**Published:** 2023-01-23

**Authors:** Natasa Vukovic, Aina Segués, Shuyu Huang, Martin Waterfall, Alice J.A.M. Sijts, Dietmar M. Zaiss

**Affiliations:** 1 Institute of Immunology and Infection Research, School of Biological Sciences, University of Edinburgh, Edinburgh, Scotland.; 2 Faculty of Veterinary Medicine, Department of Infectious Diseases and Immunology, Utrecht University, Utrecht, the Netherlands.; 3 Department of Immune Medicine, University Regensburg, Regensburg, Germany.; 4 Institute of Clinical Chemistry and Laboratory Medicine, University Hospital Regensburg, Regensburg, Germany.; 5 Institute of Pathology, University Regensburg, Regensburg, Germany.

## Abstract

In the last decades, antibody-based tumor therapy has fundamentally improved the efficacy of treatment for patients with cancer. Currently, almost all tumor antigen–targeting antibodies approved for clinical application are of IgG1 Fc isotype. Similarly, the mouse homolog mIgG2a is the most commonly used in tumor mouse models. However, in mice, the efficacy of antibody-based tumor therapy is largely restricted to a prophylactic application. Direct isotype comparison studies in mice in a therapeutic setting are scarce. In this study, we assessed the efficacy of mouse tumor-targeting antibodies of different isotypes in a therapeutic setting using a highly systematic approach. To this end, we engineered and expressed antibodies of the same specificity but different isotypes, targeting the artificial tumor antigen CD90.1/Thy1.1 expressed by B16 melanoma cells. Our experiments revealed that in a therapeutic setting mIgG2a was superior to both mIgE and mIgG1 in controlling tumor growth. Furthermore, the observed mIgG2a antitumor effect was entirely Fc mediated as the protection was lost when an Fc-silenced mIgG2a isotype (LALA-PG mutations) was used. These data confirm mIgG2a superiority in a therapeutic tumor model.

**Significance:**

Direct comparisons of different antibody isotypes of the same specificity in cancer settings are still scarce. Here, it is shown that mIgG2a has a greater effect compared with mIgG1 and mIgE in controlling tumor growth in a therapeutic setting.

## Introduction

mAbs are among the fastest-growing class of drugs, with more than 100 mAbs with marketing approval since 1986 (1). Most of them belong to cancer therapeutics (2), where their introduction critically contributed to better outcomes and increased survival for different types of cancer. However, many patients are still unresponsive to such tumor-targeting antibody therapy, underlying the need for further optimization of antibody-based approaches.

Most of the mAbs used in cancer therapy target tumor antigens which are, to varying extent, involved in tumor survival, growth, and invasiveness. Interfering with tumor cell signaling pathways can induce tumor cell death on its own (e.g., anti-HER2, anti-EGFR; refs. 3, 4). However, it has become increasingly apparent that Fc-mediated activation of the immune system substantially contributes to tumor cell destruction and the efficacy of treatment (4, 5). With their Fc tail, antibodies can engage the complement system and different effector cells such as natural killer (NK) cells and macrophages, mediating antibody-dependent cell-mediated cytotoxicity (ADCC), antibody-dependent cell-mediated phagocytosis, and complement-dependent cytotoxicity (CDC) against tumor cells (5, 6). Because different antibody isotypes bind to different FcRs on immune cells and differ in their potential to activate the complement system, they can induce diverse immune responses. Thus, the downstream effector function is determined by antibody isotype.

For murine IgG antibodies, it has been established that mIgG2a offers superior activity to mIgG1, mostly due to differential affinity for activating and inhibitory FcRs, also defined as activating-to-inhibitory (A/I) ratio. Similar to human IgG1, mIgG2a has high A/I ratio reflecting its high affinity for activating FcRs and low affinity for the inhibitory one. In contrast, mIgG1 shows very low A/I ratio (7). On the basis of the seminal publication by Nimmerjahn and colleagues (8), mIgG2a has been dominantly used as the most active antibody isotype in mouse tumor models. Here, the tumor-targeting mIgG2a showed superior tumor control to mIgG1 in B16 lung metastasis model. However, the antibody treatment in this study was prophylactic, as it started on the same day when the tumor cells were injected. On the other hand, the same antibody typically failed to control the tumor growth in a therapeutic setting once the tumors were established (9).

Therefore, the aim of this study was to compare the *in vivo* efficacy of tumor-targeting antibodies of different isotypes in a therapeutic setting. To this end, we followed a similar approach as in the prophylactic setting (8) and compared the therapeutic efficacy of one specific mAb with either a mIgG2a, mIgG1, or mIgE isotype. Our results show that mIgG2a was superior to both mIgE and mIgG1 in controlling tumor growth in a therapeutic setting. Furthermore, the observed mIgG2a antitumor effect was entirely Fc mediated as the protection was lost when an Fc-silenced mIgG2a isotype (via LALA-PG mutations) was used.

## Materials and Methods

### Antibody Design, Production, and Purification

**FIGURE 1:**
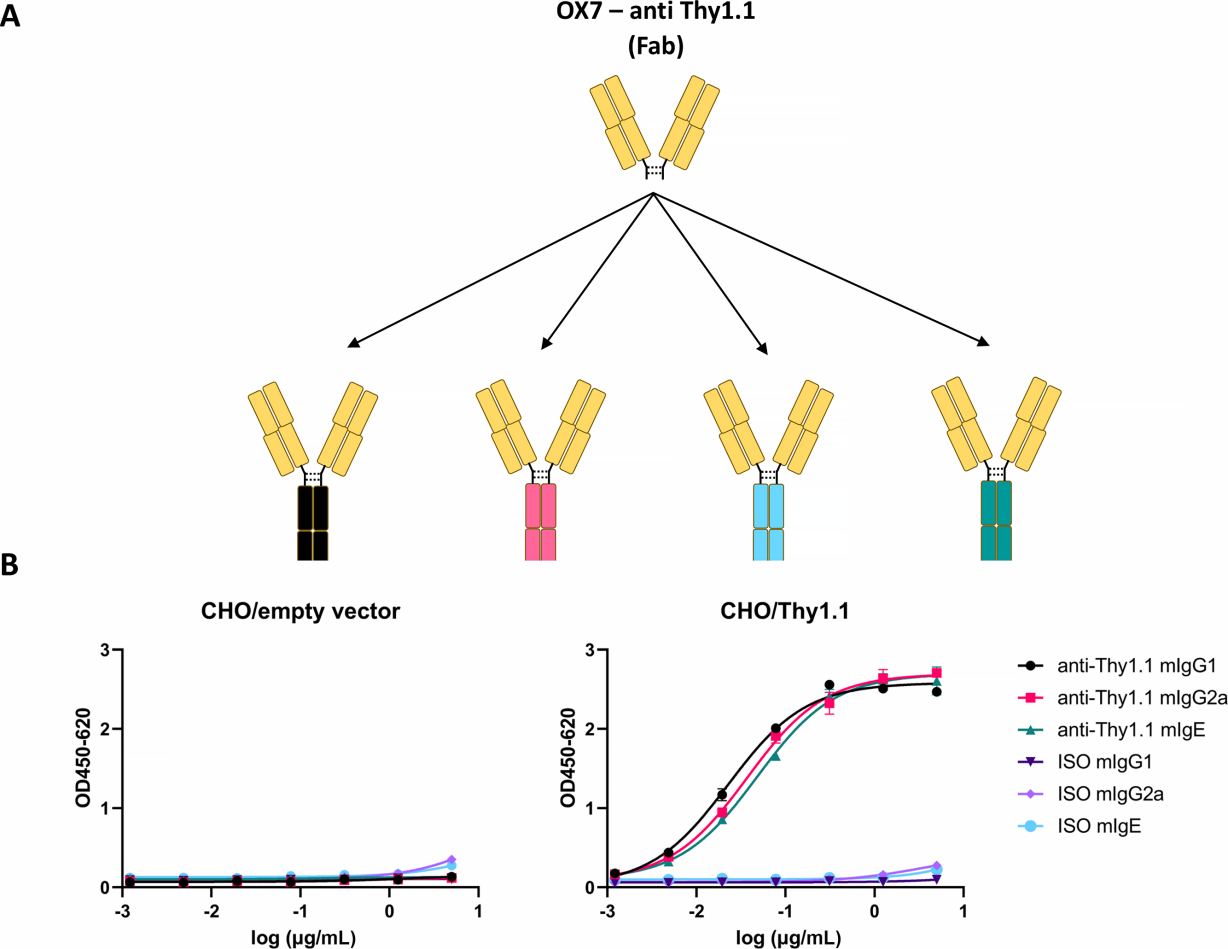
Panel of the different OX-7 antibodies targeting Thy1.1 used. **A,** Schematic summary of the different isotypes of OX7 antibodies used. Fab, fragment antigen binding. **B,** Cell binding ELISA of anti-Thy1.1 antibodies. The binding of anti-Thy1.1 IgG1, IgG2a, and IgE was tested on CHO cells transiently transfected with an empty vector (left) or Thy1.1 (right). Isotype controls were used for each antibody isotype. Mean + SD of duplicates are shown.

Amino acid sequences of all anti-Thy1.1 antibodies are provided in Supplementary Table S1. The design and production of murine anti-Thy1.1 IgG1 and IgE have been done as described before (10). In short, the starting point was OX7 hybridoma (anti-Thy1.1 IgG1) which was sequenced to obtain heavy (HC) and light chain (LC) variable domain sequences (VH, VL). Next, we designed chimeric anti-Thy1.1 mIgE and mIgG1 HCs by combining the VH with the known sequences of the constant domains of murine IgE or IgG1 (CHs). Just between VH and CH domains, a unique restriction site (AfeI) was introduced, allowing us to change the isotypes by cloning. The IgG2a HC and the IgG2a HC featuring silencing LALA-PG mutations were cloned using standard cloning techniques from plasmids available in house (anti-Siglec and anti-TNFR2, respectively) into the pcDNA3.1(+) encoding for anti-Thy1.1_VH (Fig. 1A and B). Correct clones were confirmed by Sanger sequencing (GENEWIZ). The plasmid encoding for the anti-Thy1.1 LC was *de novo* synthesized (GeneArt).

Anti-Thy1.1 IgG2a and anti-Thy1.1 IgG2a-LALA-PG were produced in ExpiCHO-S cells and FreeStyle293 cells, respectively, as described before (10). Purification was done with MabSelect SuRe LX resin. Anti-Thy1.1 IgG2a had to be polished with preparative size-exclusion chromatography (SEC). Preparative SEC and the quality control consisting of UPLC-SEC, CE-SDS, and SDS-PAGE were performed as described previously (10).

### Thy1.1 Plasmids

Full-length Thy1.1 was cloned from pCR4-Blunt-TOPO into pcDNA3.1(+) with EcoRI and ApaI two-step digestion, using a standard cloning procedure. In short, digested bands of interest were excized from the gel and extracted with Qiagen Gel Extraction Kit, according to manufacturer's protocol. Dephosphorylation of the vector and subsequent ligation were done with Rapid DNA Dephos & Ligation Kit (Roche) in 1:3 vector:insert molar ratio. DH5α competent cells were transformed with the ligation reaction and plated on LBampicillin plates. Colonies were picked, expanded, and submitted to plasmid isolation with MidiPrep Kit (GenElute HP, Sigma). The correct clone was confirmed by Sanger sequencing with T7 promoter and BGH-R universal primers (Macrogen).

Glycophosphatidylinositol (GPI) anchor of Thy1 was replaced with MHC-1 transmembrane domain in the following way. Thy1.1 propeptide, which is removed when GPI is attached to Cys130 in the endoplasmic reticulum, was replaced with a part of MHC-1 molecule (Uniprot ID P01900) consisting of the connecting peptide, transmembrane domain and cytoplasmic region. pcDNA3.1(+)_Thy1.1-MHC-1 plasmid was *de novo* synthesized (Biomatik). Thy1.1-MHC-1 was cloned into a pSG5 vector using standard cloning techniques described above with EcoRI and BglII restriction enzymes in two-step digestion. The correct clone was confirmed by Sanger sequencing (University of Dundee). The amino acid sequence of the designed construct is given in Supplementary Table S2.

### Cell Culture

The B16-OVA cells with intracellular ovalbumin (OVA) were a kind gift from Ton Schumacher (The Netherlands Cancer Institute; ref. 11). Cell line authentication was not performed, except confirming OVA expression with Western blot analysis. They were cultured in Iscove's modified Dulbecco's medium (IMDM; Gibco) supplemented with 10% heat-inactivated FBS (Gibco), 1% penicillin/streptomycin (Gibco), 2 mmol/L l-glutamine (Gibco), and 50 μmol/L 2-mercaptoethanol (Gibco; IMDM complete). CHO.K1 cells (ATCC CCL-61) were cultured in DMEM/F12 medium (Gibco) supplemented with 5% New Born Calf Serum (Biowest) and 1% penicillin/streptomycin (Gibco). No regular *Mycoplasma* testing was performed.

### Generation of B16-OVA-Thy1.1 Stable Cell Line

B16-OVA cells were cotransfected with 1.5 μg of pSG5-Thy1.1-MHC-1 plasmid and 0.5 μg of pLXSP plasmid coding for puromycin resistance with FuGENE HD reagent (Promega) in 6:1 FuGENE: DNA ratio. Briefly, the DNA was diluted in OptiMEM medium, after which FuGENE HD was added, and the mixture was incubated for 10 minutes at room temperature. The transfection mixture was added dropwise to the cells at 80% confluency. A total of 24 hours after transfection, 3 μg/mL of puromycin was added to the culture medium, and the cells were grown under puromycin pressure for 10–14 days. Selected cells were stained with 2 μg/mL of PE anti-Thy1.1 antibody (OX7 clone, BioLegend #202524) and single-cell sorted into 96-well plates containing the selection medium with puromycin. Thy1.1 expression was regularly monitored by flow cytometry with the antibody mentioned above on FACSCanto. Positive clones were expanded and the one showing stable Thy1.1 expression even after puromycin retrieval was selected for the *in vivo* study.

### Thy1.1 Transient Transfection and Cell ELISA

An amount of 24 μg of pcDNA3.1(+)-Thy1.1 plasmid was transfected into CHO.K1 cells (10 mm Petri dish, 80% confluent) using the lipofectamine 2000 reagent (Invitrogen) according to the manufacturer's recommendation. The following day, cells were plated into a 96-well plate (5 × 10^5^ cells/well). Two days after transfection, an antibody binding ELISA was performed. The cell supernatant was discarded, and either anti-Thy1.1 IgE, IgG2a, or IgG1 were added in serial dilutions. After incubation at room temperature for 1 hour, goat anti-mouse IgE-HRP conjugate (Southern Biotech, 1:4,000) or goat anti-mouse IgG Fc-HRP (Jackson Immuno Research 1:5,000) in 1:1 1% BSA phosphate-buffered saline with Tween 20 (PBS/PBST) were added for 45 minutes at room temperature. Immunoreactivity was visualized with TMB Stabilized Chromogen (Invitrogen). Reactions were stopped after 15 minutes with 0.5 mol/L H_2_SO_4_, and absorbances were read at 450 and 620 nm. All samples were tested in duplicate.

### OT-1 Activation

Fresh spleens from OT-1 mice were used for splenocyte isolation. The spleens were mashed through a 70 μm cell strainer, after which the Red Blood Cell (RBC) lysing Buffer (Hybri-Max, Sigma) was used to remove any erythrocytes. The splenocytes were plated at the density of 0.5 million cells/mL in 12-well plates (1 mL/well). They were cultured in IMDM (Gibco) supplemented with 10% heat-inactivated FBS (Gibco), 1% penicillin/streptomycin (Gibco), 2 mmol/L l-glutamine (Gibco), 50 μmol/L 2-mercaptoethanol (Gibco), and 2 μg/mL OVA peptide (SIINFEKL). A total of 48 hours later (day 2), the cells were subcultured 1:2. On day 3, the activated OT-1 cells were washed with PBS and injected intravenously via tail. OT-1 activation was confirmed by flow cytometry based on CD8 (BD Biosciences) and CD25 (BioLegend) expression using FACS anlysis. Consistently we found that about 90% of the cells injected were fully activated OT-1 (CD8^+^ CD25^+^; Supplementary Fig. S3A).

### Mice

OT-1 mice were maintained in the animal facility at the University of Edinburgh (Edinburgh, Scotland). Age-matched, 6–10 weeks old female mice on a C57BL/6 background were purchased from Charles River. Experiments were carried out under the project license PPL: PP7488818. All animal experiments were approved by The University of Edinburgh (Edinburgh, Scotland).

### Tumor Rejection Studies

After thawing, B16-OVA-Thy1.1-MHC1 cells were cultured for about a week (∼3 passages) before injecting into mice. A total of 5 × 10^5^ B16-OVA-Thy1.1-MHC-1 cells were subcutaneously injected into the right flank. Antibody treatment consisted of either 200 μg anti-Thy1.1 IgG2a or 200 μg anti-Thy1.1 Ig1 or 10 μg anti-Thy1.1 IgE (all in house produced as described above). IgGs were administered intraperitoneally, whereas IgE was administered intravenously. The antibodies were injected on days 7, 13, 17, and 24. Some mice received the adoptive cell transfer (ACT) of 2.5 × 10^5^ activated OT-1 cells in PBS intravenously on day 13. The tumor size was measured regularly with a calliper. The mice were sacrificed when the tumors reached 10 mm in diameter or at the first sign of ulceration or if significant weight loss was observed (>20% of initial weight). Tumor volume was calculated by the modified ellipsoidal formula: *V* = ½ (length × width^2^).

### CDC Assay

B16-OVA and B16-OVA-Thy1.1 cells were detached with 2 mmol/L Ethylenediamine tetraacetic acid (EDTA; Gibco) and were prestained with eF450 and eF670 (eBioscience) respectively, following manufacturers’ instructions. The stained cells were then mixed in 1:1 ratio in 96-well round bottom plate (5 × 10^5^ cells per well). Cells were washed three times with FACS buffer (1% FBS in PBS) at 400 × *g* for 3 minutes at 4°C and incubated with indicated antibodies at 50 μg/mL (50 μL/well) for 30 minutes at 4°C in the dark. Next, the cells were washed three times and were incubated with prewarmed Rabbit Complement (RC; Cedarlane) diluted 1:8 in IMDM complete media (50 μL of RC/well). The cells were incubated for 1 hour at 37°C, after which DNAse (Promega; 1 U/μL) diluted in FACS buffer was added and the cells were washed three times. Finally, the cells were resuspended in 150 μL FACS buffer with 1 mg/mL propidium iodide (PI; Sigma-Aldrich). A total of 100 μL of the stained cells were analyzed on a FACS LSRFortessa (BD) using the software program BD FACSDiva. Further analysis was performed with FlowJo and shown results plotted in GraphPad.

### Generation of NK Cells

Spleens from Rag1 knockout mice were homogenized and submitted to RBC lysis using the RBC lysis buffer (Sigma-Aldrich). The splenocytes were seeded at 2 × 10^6^ cells/mL in 24-well plates with RPMI (Sigma) supplemented with 10% heat-inactivated FBS (Gibco), 1% penicillin/streptomycin (Gibco), 2 mmol/L l-glutamine (Gibco), 50 μmol/L 2-mercaptoethanol (Gibco), 20 ng/mL of IL2 (BD Pharmingen), and 20 ng/mL of IL15 (Peprotech). Cells were used at day 5 when approximately 95% of intact cell population was identified as NK cells based on the expression of NKp46 (eBioscience) and NK1.1 (eBioscience) and lack of expression of CD3 (BD Pharmingen) by flow cytometry (CD3^−^ NKp46^+^ NK1.1^+^) using FACS LSRFortessa (BD).

### ADCC Assay

B16-OVA and B16-OVA-Thy1.1 target cells were detached with 2 mmol/L EDTA (Gibco) and added to 96-well round bottom plates at 1 × 10^4^ cells/well. The indicated anti-Thy1.1 antibodies were added at 10 μg/mL/well in FACS buffer and incubated for 30 minutes at 4°C, followed by two washing steps with FACS buffer at 400 × *g* for 3 minutes at 4°C. The live effector NK cells were counted using trypan blue staining and a viability of about 95% was consistently observed. NK cells were then added in prewarmed media at 3-fold decreasing concentrations starting at 9:1 effector:target ratio. The cells were centrifuged at 400 × *g* for 2 minutes to concentrate them at the bottom of the wells and ADCC assay was run for 4 hours at 37°C. After 4 hours of incubation, the cells were centrifuged at 300 × *g* for 5 minutes, and the supernatant was used to assess the cell toxicity with CytoTox 96 Non-Radioactive Cytotoxicity Assay lactate dehydrogenase (LDH) cytotoxicity Assay kit (Promega) following manufacturer's instructions. The LDH activity of medium alone was subtracted from the LDH activity of test conditions to obtain the corrected values. These corrected values were then used to calculate the percentage of cellular cytotoxicity using the following formula: percentage specific lysis 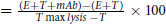, where E are the effector cells, T are the target cells, and Tmax the lysed target cells alone.

### Statistical Analysis

Statistical analysis was performed in GraphPad Prism software. Survival was evaluated with the Mantel–Cox test. *P* values of ≤0.05 were considered statistically significant. ns, *P* > 0.05; *, *P* ≤ 0.05. CDC assay was evaluated by one-way ANOVA applied to subtracted values (no RC − with RC) of each condition. ADCC assay was evaluated by multiple *t* test at each specific ratio. Indicated * mean the significant difference between B16-OVA-Thy1.1 IgG1 and IgG2a versus all the other conditions.

### Data Availability

Data were generated by the authors and included in the article. The data generated in this study are available within the article and its Supplementary Data. Raw data are available upon request from the corresponding author.

## Results

### Expression of Anti-Thy1.1 Antibodies with Different Fc isotypes

To compare different isotypes in a therapeutic setting, we repeated an approach used by Nimmerjahn and colleagues (8) and expressed antibodies with the same specificity but different isotypes (Fig. 1A). For our study, we chose an antibody, which recognizes CD90.1/Thy1.1, a congenic marker often used for immunologic studies. This antibody binds to lymphocytes expressing Thy1.1, which is expressed by some mouse lines, such as AKR mice, but does not bind to Thy1.2, which is expressed by other mouse lines, such as C57BL/6. To this end, we sequenced the HC and LC variable domain sequences (VH, VL) of the OX7 hybridoma (anti-Thy1.1). OX7 expresses antibodies with an IgG1 isotype and is known to lack cell-depleting activity once injected into mice. We therefore designed chimeric anti-Thy1.1 mIgG2a HCs by combining the VH with the known sequences of the constant domains of murine IgG2a (CHs). In addition, we expressed antibodies with the same anti-Thy1.1 specificity but an IgE isotype. This was mainly due to the fact that in some preclinical models, IgE antibodies have been shown to exhibit superior tumor control in comparison with their IgG homologs (12, 13).

The anti-Thy1.1 antibodies with different Fc isotypes were expressed *in vitro* and purified using MabSelect SuRe LX resin. Preparative SEC and quality control consisting of ultra-performance liquid chromatography (UPLC)-SEC, capillary electrophoresis SDS (CE-SDS), and SDS-PAGE were performed. Size-exclusion UPLC showed that all three antibodies (anti-Thy1.1 IgG1, IgG2a, and IgE) reached monomericity levels of >95% (Supplementary Fig. S1A). Next, the purity was tested by CE-SDS. Because CE-SDS was not optimized for IgE, we also included SDS-PAGE to confirm the correct molecular weights and purity of IgE. The analysis under nonreducing conditions confirmed the expected molecular weights and indicated that a high purity (>90%) was reached in all samples [Supplementary Fig. S1B (left) and S1C]. Furthermore, only HC and LC were observed under reducing conditions, confirming the correct sample composition [Supplementary Fig. 1B (right) and S1C].

Taken together, the produced antibodies complied with high-quality standards regarding monomericity and purity. In addition, we confirmed that the antigen binding was preserved in binding ELISA with Thy1.1-expressing CHO cells (Fig. 1B). Importantly, no difference in binding was observed between different isotypes.

### Stable Thy1.1 Expression by B16-OVA Cells

**FIGURE 2:**
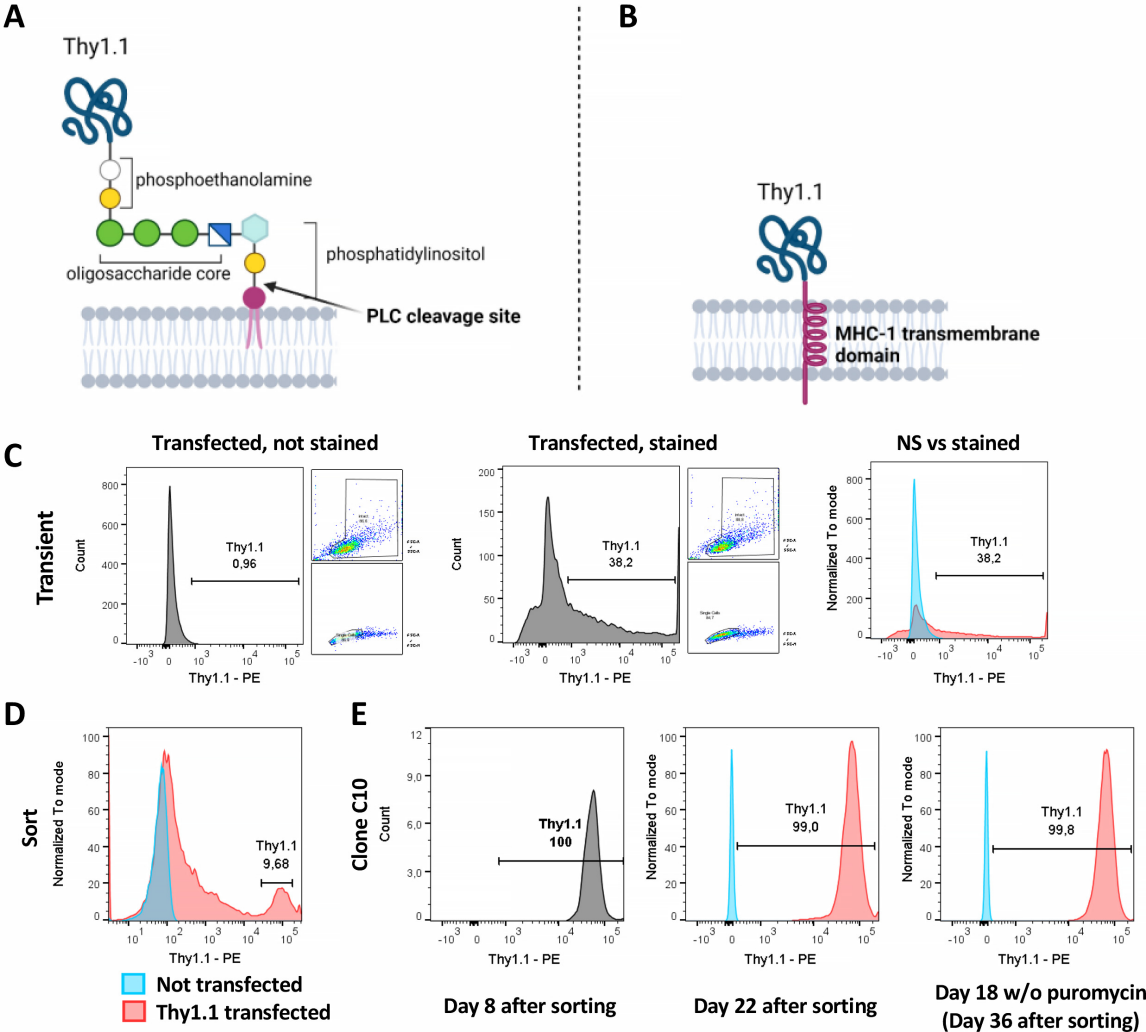
Thy1.1-MHC-1 expression on B16-OVA cell. B16-OVA cells were cotransfected with pSG5-Thy1.1-MHC-1 and pLXSP, selection agent (puromycin) was added 24 hours after transfection and single-cell sorting was performed after at least 10 days of growing the cells in the selection medium. Thy1.1 expression was regularly tested by FACS. Schematic representation of Thy1.1 with its GPI anchor (**A**) and the designed construct in which the GPI anchor has been replaced with MHC-1 transmembrane domain (**B**). **C–E,** FACS analysis of Thy1.1 expression on B16-OVA cells after transfection with pSG5-Thy1.1_MHC-1. **C,** Transient expression 24 hours after transfection. **D,** Expression at single-cell sorting. **E,** Expression on the selected clone on the indicated days.

CD90 (Thy1) is a GPI-anchored cell surface protein, and it is, therefore, susceptible to the cleavage of GPI anchor by Phospholipase-C (ref. 14; Fig. 2A). To overcome a possible loss of expression, as it has been reported before (15), we replaced the GPI anchor of Thy1.1 with a murine MHC-1 transmembrane domain (Fig. 2B). Transfected B16-OVA cells were tested for their expression stability for about 5 weeks. B16-OVA-Thy1.1 clone showed no changes in Thy1.1 expression even after removal of puromycin used for selection, confirming stable expression by this clone (Fig. 2C–E). The replacement of the Thy1.1 transmembrane domain did not affect the binding capacity of anti-Thy1.1 antibodies, as Thy1.1-MHC-1 expression levels were measured using the same anti-Thy1.1 antibody clone (OX7).

### Different CDC and ADCC Profiles for IgG2a, IgG1, and IgE Antibodies

**FIGURE 3:**
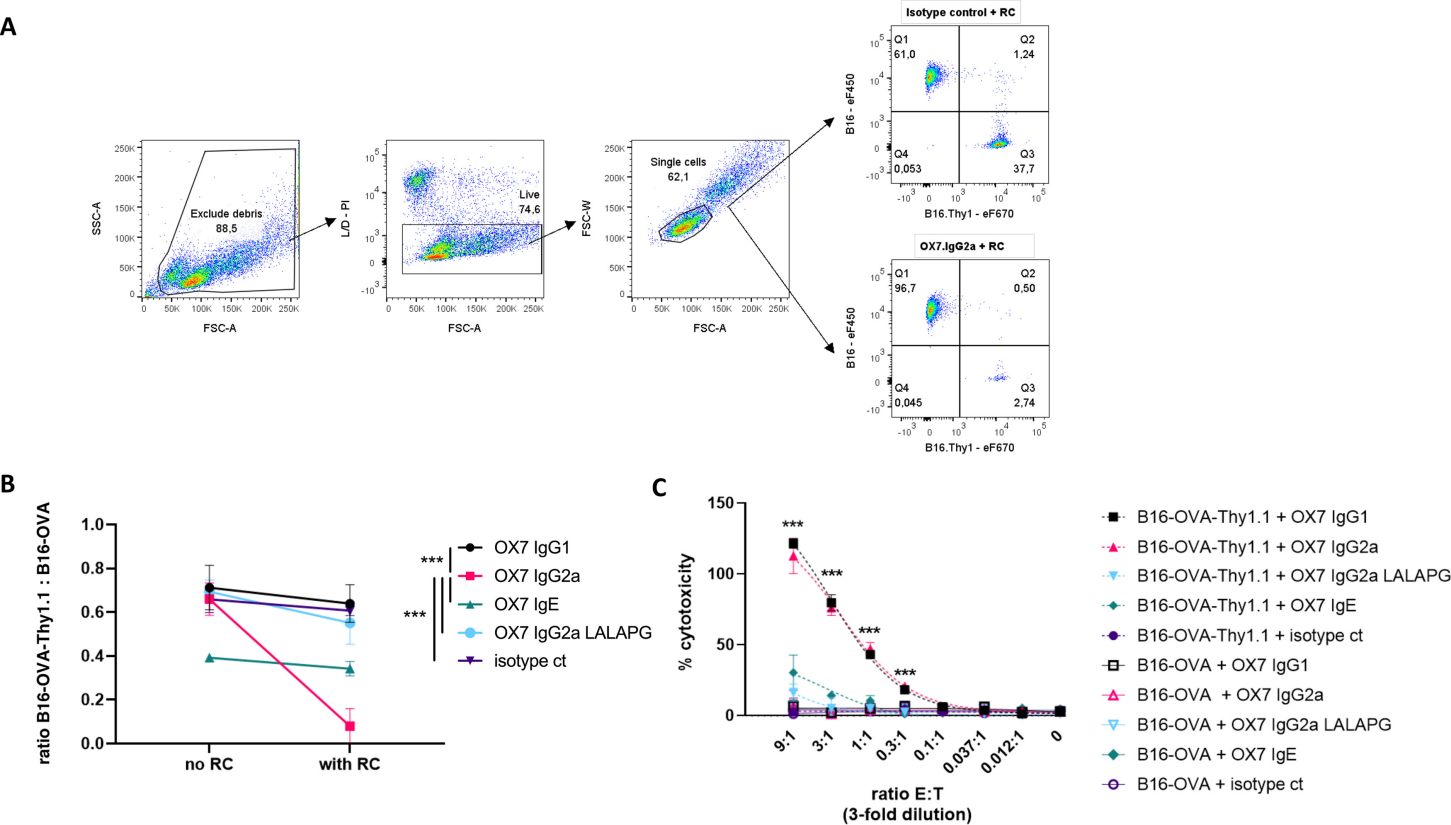
CDC and ADCC profiles of anti-Thy1.1 IgG1, IgG2a, IgE, and IgG2a-LALA-PG. **A,** Representative plots used to calculate B16-OVA-Thy1.1:B16-OVA ratio. First, B16 cells were gated based on FSC-A/SSC-A properties. Next, Live cells were based on FSC-A/PI staining. Live cells were gated for single cells based FSC-A/FSC-W. Target cells B16-OVA-Thy1.1 are found in Q3 as eF670+ and B16-OVA are found as Q1 as eF450+. Data representative from samples incubated isotype control or OX7.IgG2a and with RC. **B,** B16-OVA-Thy1.1 target cells and B16-OVA control cells were previously stained, then coincubated with 50 μg/mL of anti-Thy1.1 antibodies at 4°C for 30 minutes and finally incubated with RC for 1 hour at 37°C. Cells were analyzed by FACS and B16-OVA-Thy1.1:B16-OVA ratio was calculated. **C,** B16-OVA-Thy1.1 target cells and B16-OVA control cells were incubated independently with 10 μg/mL of anti-Thy1.1 antibodies and then coincubated at various effector-to-target ratios with NK cells for 4 hours at 37°C. CytoTox 96 Non-Radioactive Cytotoxicity Assay LDH cytotoxicity Assay kit was used to assess cytotoxic effect mediated by the antibodies. Mean + SD of triplicates are shown of a representative biological replicate out of *n* = 3 biological replicates. [Statistics: CDC assay—one-way ANOVA on subtracted values (no RC − with RC); ADCC assay—multiple *t* test, ***, *P* < 0.001].

To assess the capacity of the different antibodies to induce complement-mediated CDC and NK cell–mediated ADCC, *in vitro* cytotoxicity assays were performed. To detect on-target CDC killing, we mixed B16-OVA-Thy1.1 target cells with B16-OVA control cells in 1:1 ratio and tested how the ratio changes after antibody-mediated complement activation. As expected, only IgG2a significantly reduced the ratio (Fig. 3A and B), suggesting that only the IgG2a isotype successfully mediated CDC against target cells. Furthermore, as a control, the introduction of the Fc-silencing LALA-PG mutations into IgG2 isotype abrogated the complement-mediated activity (Fig. 3B). In parallel, different antibody isotypes were evaluated in an ADCC assay where NK cells were used as effector cell population (Supplementary Fig. S2). Here, both IgG2a and IgG1 showed high cytotoxicity toward B16-OVA-Thy1.1 cells (Fig. 3C), whereas IgE and IgG2a-LALA-PG did not induce NK cell–mediated cell killing. Finally, no cytotoxicity was observed with B16-OVA control cells not expressing Thy1.1 antigen with any of the tested isotypes.

Taken together, these data show that the expressed antibodies retained their described effector function. Although our data showed the highest complement-mediated activity for IgG2a, the ADCC effect was similar for both IgG2a and IgG1. This is to be expected as NK cells were used as effector cells in the ADCC assay. NK cells only express FcγRIII (16, 17), which shows similar binding profiles for IgG1 and IgG2a (18). Nonetheless, IgG2a presents higher affinity for the activating FcγRIV, which is absent on NK cells, but present on macrophages. Therefore, *in vivo*, where macrophages may also contribute as effector cells, superior effector function of IgG2a-expressing antibodies could be postulated (19–21).

### IgG2a Antibodies Show Superior Therapeutic Tumor Control to Their IgG1 and IgE Homologs

**FIGURE 4:**
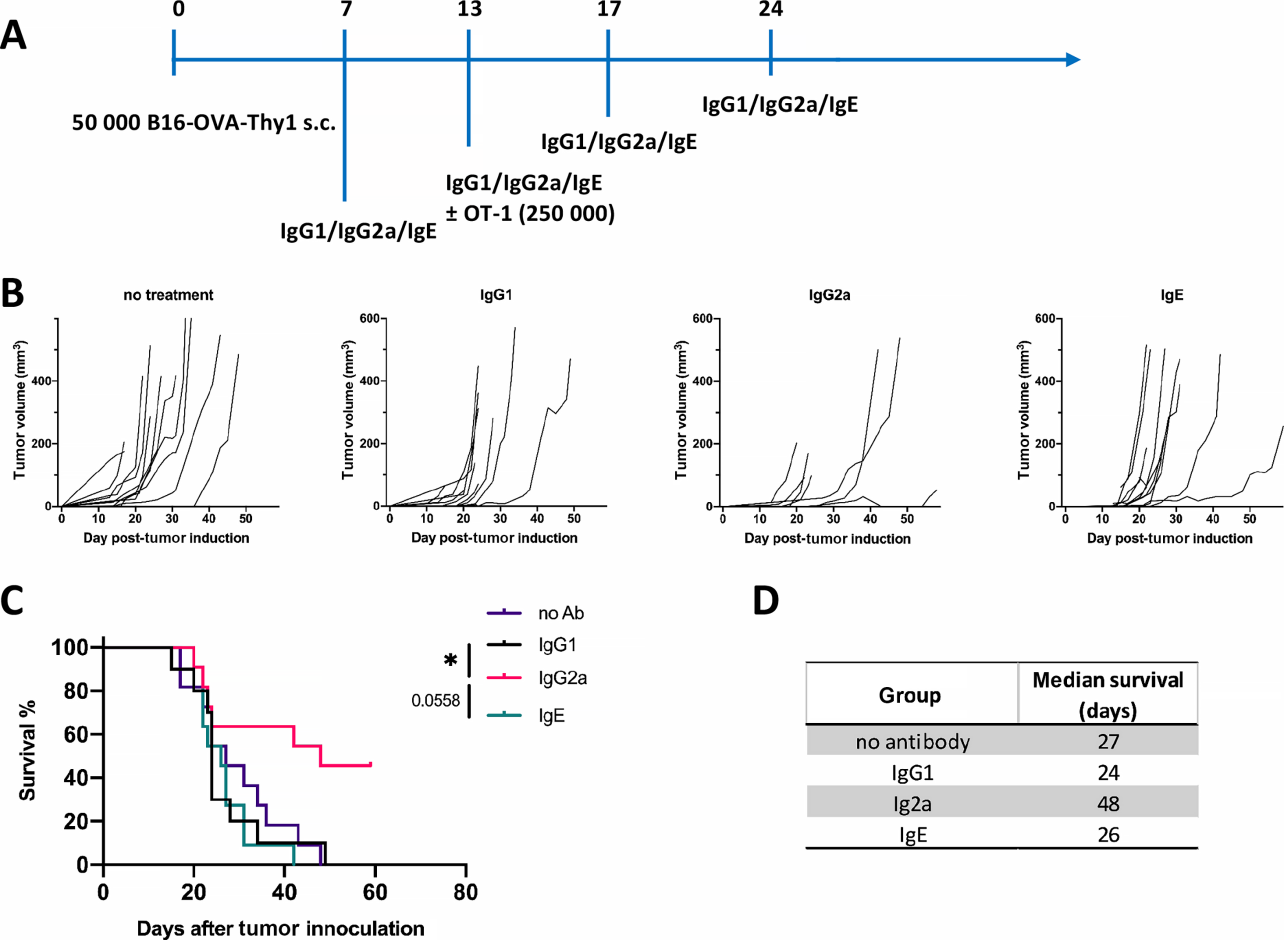
Superior tumor growth control of anti-Thy1.1 IgG2a *in vivo*. C57BL/6 mice were subcutaneously injected with 50 000 B16-OVA-Thy1.1 cells in the flank and were treated with anti-Thy1.1 IgG1, IgG2a, or IgE antibodies. **A,** Experimental scheme of the antibody isotype comparison in the B16-OVA-Thy1.1 model. **B,** Tumor growth curves. **C,** Survival analysis. **D,** Median survival in days. Statistical significance was calculated with the Mantel–Cox test. *, *P* ≤ 0.05. **B–D**: *n* = 10–12, combined data of two independent experiments.

To test the therapeutic capacity of different antibody isotypes to control tumor growth in a syngeneic mouse model, C57BL/6 mice were subcutaneously injected with B16-OVA-Thy1.1 cells and treated with either anti-Thy1.1 IgG2a, IgG1, or IgE antibodies, starting on day 7 after tumor cells transfer (Fig. 4A). Similar to the prophylactic setting, in this therapeutic setting antibody treatment with an IgG2a isotype showed superior tumor growth control compared with antibodies with an IgG1 or IgE isotype (Fig. 4B and C). Whereas all IgG1-treated (10/10) or IgE (12/12)-treated animals reached the human-defined endpoint by day 49, 50% (6/12) of IgG2a antibody–treated mice showed very small or no tumor growth at all, at day 60. Median survival was 24 days for IgG1 and 26 days for IgE, compared with 48 days for IgG2a (Fig. 4D).

**FIGURE 5:**
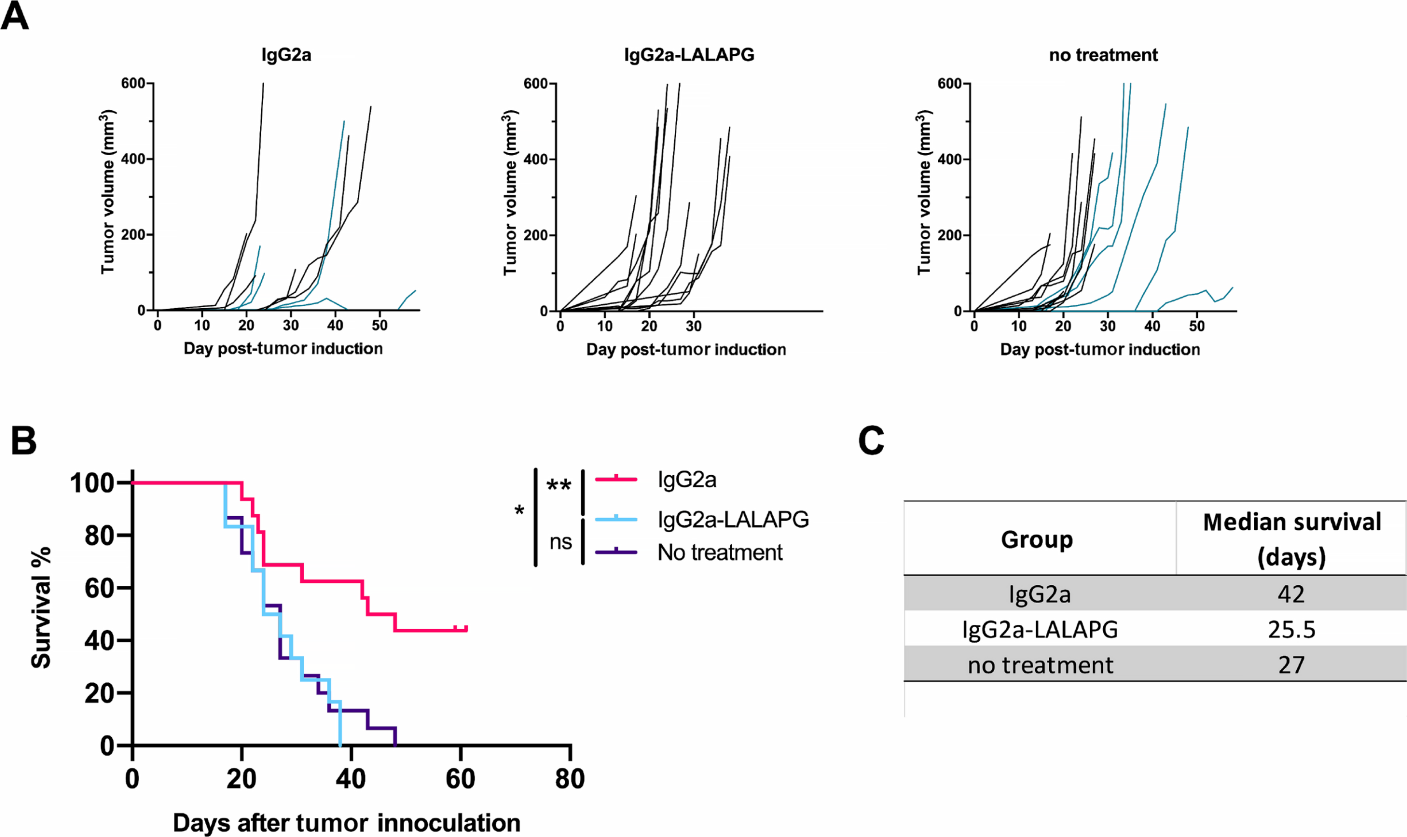
*In vivo* tumor control is lost when IgG2a Fc tail is silenced. C57BL/6 mice were subcutaneously injected with 50 000 B16-OVA-Thy1.1 cells in the flank and were treated with anti-Thy1.1 IgG2a (active) or anti-Thy1.1 IgG2a-LALA-PG (Fc silent) antibody. **A,** Tumor growth curves. **B,** Survival analysis. **C,** Median survival in days. Combined data of three independent experiments are shown (*n* = 12–16). Blue lines in A are indicative of data from Fig. 4 in IgG2a and control group. Statistical significance was calculated with the Mantel–Cox test (*, *P* < 0.1; **, *P* < 0.01).

To confirm that the superior tumor control is mediated via the IgG2a interaction with the immune system, we introduced LALA-PG mutations in the constant domain of the IgG2a HC. LALA-PG mutations have been shown to significantly reduce the binding of both human and murine IgG antibodies to Fcγ receptors (22). In the case of mIgG2a, the binding to FcγRI, II, and IV is completely interrupted, while the binding to FcγRIII is reduced more than 50-fold. In addition, LALA-PG mutants show decreased C1q binding and C3 fixation in murine serum and, consequently, lose the capacity to mediate complement-mediated cell lysis. When we compared the anti-Thy1.1 IgG2a and IgG2a-LALA-PG *in vivo*, we observed a complete loss of efficacy with the Fc-silenced antibody (Fig. 5A). Whereas IgG2a survival rate was around 50% at day 60, all mice treated with IgG2a-LALA-PG reached the endpoint by day 39 (Fig. 5B). Median survival was 42 days for IgG2a compared with 25,5 days for IgG2a-LALAPG and 27 days for the untreated group (Fig. 5C). These results clearly show that the observed antitumor effect of the anti-Thy1.1 IgG2a antibody was Fc mediated and isotype dependent.

### Antibody Treatment is not Synergizing with T Cell–based ACT

In addition, the antibodies were also tested in combination with the ACT of activated OT-1 cells. B16-OVA tumors are characterized by an immune-suppressive tumor microenvironment (TME) dominated by regulatory T cells (T_reg_). It has been shown that depletion of intratumoral T_regs_ offers tumor protection when combined with the GVAX vaccine due to enhanced activation of CD8^+^ T cells (23, 24). These data suggest that, in this setup, OT-1 efficacy can be inversely correlated with T_reg_ function. With the B16-OVA cell line that we used, OT-1 monotherapy is usually ineffective when given after day 7 after tumor implantation. Therefore, we injected the OT-1 cells at a later stage of tumor development when they can no longer control the tumor growth due to an established immune-suppressive TME. This allowed us to test whether our antibodies attenuate this immune-suppressive TME and may rescue OT-1 efficacy. Nonetheless, our results show that OT-1–treated mice had similar outcomes to those that did not receive OT-1 ACT (Supplementary Fig. S4A). These data suggest that none of the IgG2a, IgG1, or IgE treatments synergized with ACT treatment.

## Discussion

In mice, the efficacy of antibody-based treatments is largely restricted to a prophylactic application, but lack efficacy in a therapeutic setting, once the tumor has been established. In this study, we directly compared the therapeutic activity of murine IgG2a, IgG1, and IgE antibodies of the same specificity, targeting a surface tumor antigen (Thy1.1). Wild-type mice bearing syngeneic B16-OVA-Thy1.1 tumors were used for this purpose. Our results show that in this setting antibodies with an IgG2a isotype offer superior tumor control in comparison with antibodies with an IgG1 or IgE isotype. The observed effect was entirely Fc-mediated as it was completely lost using IgG2a featuring Fc-silencing LALA-PG mutations.

IgG2a is known as the most active IgG subclass in mice due to its high A/I ratio. Nevertheless, direct comparisons of different antibody isotypes of the same specificity in cancer settings are still scarce, although the first mechanistic basis for different activity of IgG subclasses was provided in 2005 (8). By using the B16-F10 lung metastasis model and a prophylactic treatment with TA99 antibody of different IgG subclasses (targeting Trp1 expressed on B16-F10 cells), the authors showed in that study that IgG2a offers superior tumor control to IgG1, IgG2b, and IgG3 (8). However, these TA99 antibodies lack activity in a therapeutic setting (9). Furthermore, Dahan and colleagues showed that an anti-PD-L1 IgG2a antibody is superior to IgG1 in MC38 and B16-OVA tumor models (25). However, PD-L1 expression is not restricted to tumor cells and has a substantial influence on local immune responses within tumors, making it challenging to extrapolate these results to exclusively tumor antigen–targeting mAbs.

Here, we sought to further our understanding of the therapeutic capacity of IgG2a-expressing antibodies. To this end, we focused our study exclusively on therapeutic setting and started antibody-based treatment on day 7 after tumor cell injection. Furthermore, we focused our study on an artificial and well-characterized model antigen exclusively presented by tumor cells. For this purpose, Thy1.1 was chosen as a target antigen. As wild-type C57BL/6 mice express only Thy1.2, the anti-Thy1.1 antibody treatment is tumor selective. Furthermore, in contrast to other model tumor antigens, Thy1.1 has not functional importance for the tumor cell as such. Therefore, the antitumor effect observed is solely due to Fc-mediated effects, making it an ideal model system for comparing the therapeutic efficacy of different antibody isotypes.

In addition, we also included antibodies expressing the IgE isotype in this study. In multiple preclinical studies, antibodies with the IgE isotype have been shown to mediate superior antitumor effects in comparison with antibodies expressing commonly used IgG isotypes (12, 13, 26). However, these studies have not addressed the potential outcome of IgE-mediated activation of mast cells (MC) and basophils on tumor development. Because IgE can induce extremely potent immune reactions through these cell types, diverting them against tumor cells could have therapeutic benefits. Mice represent a good model for addressing this question, as their FcεRI expression is limited to MCs and basophils (27). Nonetheless, our results show that IgE treatment did not have any effect on tumor growth, as the growth curves and survival rate of IgE antibody–treated mice were not significantly different compared with untreated mice. A similar approach has been recently used by a group at Massachusetts Institute of Technology that showed that IgE targeting a surface tumor antigen could not successfully control the tumor growth in B16-OVA and MC-38 models in C57BL/6 wild-type mice (28). In many studied types of tumor, MCs have been detected to be located mainly in the peritumoral and less so in the intratumoral space (29). Therefore, a lack of effect as we observed it with IgE-based antibody treatment could potentially be explained by a poor presence of IgE effector populations within B16-OVA tumors. Thus, targeting a surface tumor antigen with an IgE antibody may not be optimal for MC/basophil activation. Such limitations could potentially be overcome by using soluble tumor antigens, as they may have a higher probability of reaching MCs at the tumor edges. In line with such an assumption, our data may suggest that a tumor resident cell surface antigen, such as Thy1.1 we used in our model system, might not be an optimal IgE target for inducing MC and basophil activation at the site of solid tumors. Therefore, to perform a proper comparison between the therapeutic capacity of antibodies with an IgG2a and an IgE isotype, studies using mice with a humanized expression pattern of the IgεR (12, 13, 26) appear warranted.

Finally, we combined antibody treatment with OT-1 ACT, which, as monotherapy, is usually not effective in rejecting already established B16-OVA tumors due to the immune-suppressive TME of the tumor (11). To our knowledge, such combination therapies consisting of tumor-targeting antibodies and adoptively transferred CTLs have not been previously tested. However, they could potentially have a beneficial effect, if the antibody treatment could attenuate the immune-suppressive state of the TME. We were particularly interested, whether IgE could mediate such an effect by inducing the T_reg_ suppression via histamine released from degranulating MCs (30). Nonetheless, none of the tested antibody isotypes was able to improve the efficacy of OT-1 treatment, not even treatment with the IgG2a antibody which showed substantial efficacy in monotherapy. Such findings indicate that the immune-suppressive tumor microenvironment within the transferred B16 tumors may not have been substantially altered by the antibody treatment.

Nonetheless, one should keep in mind that such a lack of response as we have observed it in our study might not necessarily be generalizable. We purposely chose the well-established B16 melanoma model system for our study, as it allowed us to keep all other factors stable, but selectively manipulate exactly one variable, that is, the isotype of the HC of the used antibodies. However, using such a highly artificial model system also has its limitations, as other tumor models might potentially be more susceptible to antibody-mediated shifts in the TME. B16 melanoma, for instance, are not particularly susceptible to PD-1–targeted antibody treatment, while the colon carcinoma cell line MC38 is highly responsive to such treatment. Therefore, it might be worthwhile to investigate susceptibilities of different tumor models to ACT in combination with therapeutic antibody treatment in future studies. Furthermore, it appears necessary to aim for a better understanding of how such combined treatment might influence immune cell influx. Because of technical limitations, we could not assess such differences following the treatment with different antibodies in this study. However, there has been substantial progress in the field of highly sensitive techniques that might allow to explore this aspect in future studies. As mentioned before, in particular with respect to IgE antibodies such studies might be able to open entire novel fields of research and, potentially, therapeutic treatment opportunities. Alternatively, synergisms between tumor-targeting antibody treatment and T_reg_-depleting antibodies might want to be explored in more detail. In the B16 melanoma model system, it has been shown that targeting intratumoral T_regs_, using CTLA-4 antibodies, offers tumor protection when combined with CD8 T-cell inducing vaccination (23, 24). Therefore, at this stage, it remains tempting to speculate that in future experiments a combination of T_reg_-depleting or TGFβ-neutralizing antibody treatments with tumor antigen–targeting antibodies may show synergistic effects in reverting an immunosuppressive TME and, hence, in enhancing the efficacy of treatment.

Therefore, in conclusion, while this study provides *in vivo* evidence that tumor antigen–targeting IgG2a is superior to its IgG1 and IgE homologs in controlling the tumor growth in a therapeutic setting in wild-type C57BL/6 mice, future studies may have to dissect how these different isotypes influence immune cell influx into tumors and gauge their capacity to influence the immunosuppressive microenvironment within tumors.

## Supplementary Material

Supplementary Figure SF1 Quality control of anti-Thy1.1 antibodies.

Supplementary Figure SF2 Flow cytometry data used for ADCC.

Supplementary Figure SF3 Confirmation of OT-1 cells activation by flow cytometry.

Supplementary Figure SF4 No synergistic effected is achieved combining anti-thy1.1 antibodies with adoptive cell transfer of activated OT-1s.

Supplementary Table ST1 Amino acid sequences of anti-Thy1.1 antibodies.

Supplementary Table ST2 Amino acid sequence of designed Thy1.1-MHC-1 construct
