# Supplementary material for: Mouse IgG2a Isotype Therapeutic Antibodies Elicit Superior Tumor Growth Control Compared with mIgG1 or mIgE
Source: Cancer Res Commun. 2023 Jan 23;3(1):109–18. doi: 10.1158/2767-9764.CRC-22-0356 (PMC10035513; doi:10.1158/2767-9764.CRC-22-0356)
Supplement: Supplementary Figure SF1 — Quality control of anti-Thy1.1 antibodies. [file crc-22-0356-s01.pdf]

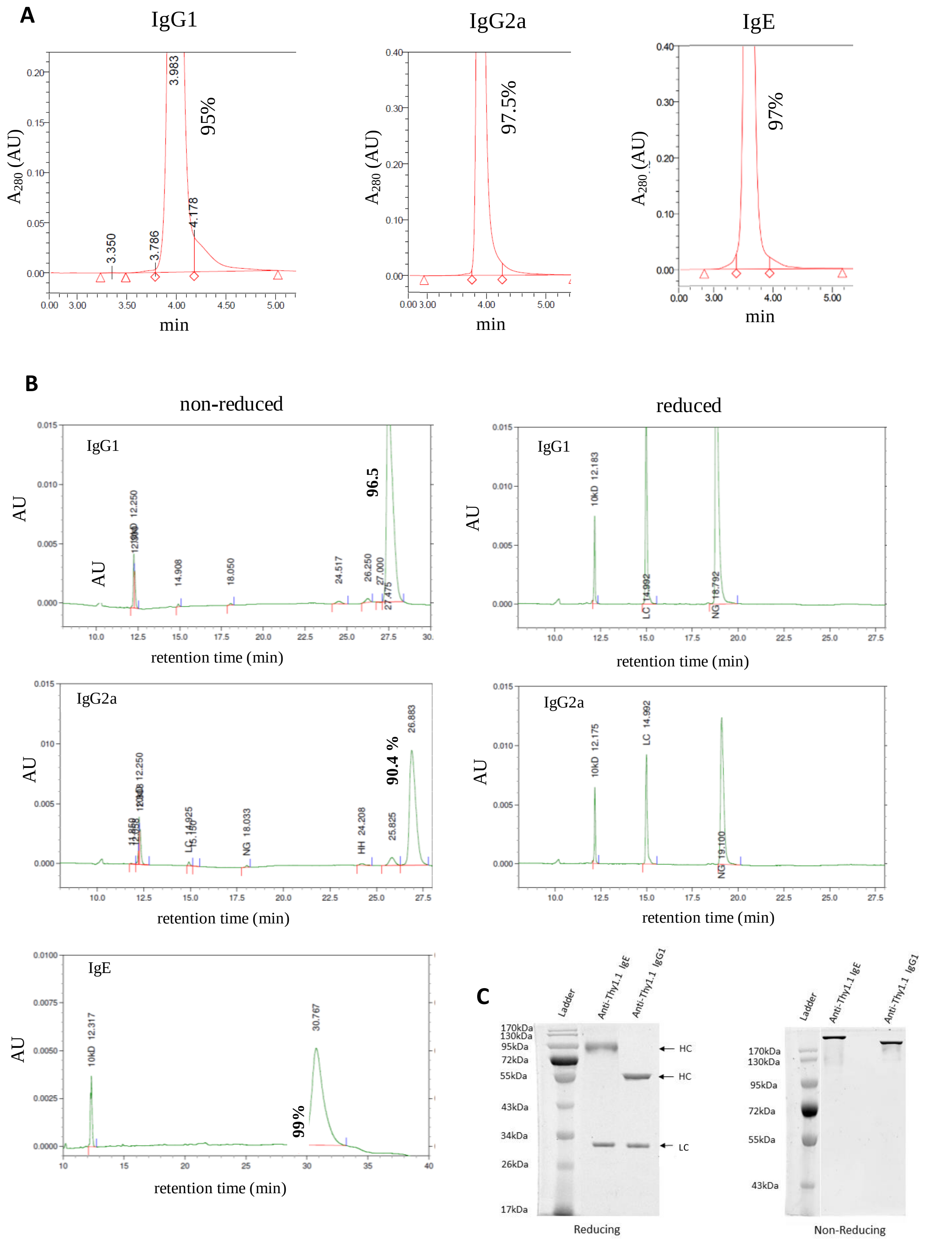

Supplementary Figure 1. Quality control of anti-Thy1.1 antibodies. (A) Monomericity was evaluated with UPLC-SEC, monomer percentage is shown; (B) CE-SDS under non-reducing conditions and purity percentage (left) and under reducing conditions (right); (C) SDS-PAGE was used for IgE evaluation as a

complementary method, since CE-SDS was not optimised for IgE. The data for IgE has been previously published.[10]
