# Supplementary material for: Mouse IgG2a Isotype Therapeutic Antibodies Elicit Superior Tumor Growth Control Compared with mIgG1 or mIgE
Source: Cancer Res Commun. 2023 Jan 23;3(1):109–18. doi: 10.1158/2767-9764.CRC-22-0356 (PMC10035513; doi:10.1158/2767-9764.CRC-22-0356)
Supplement: Supplementary Figure SF2 — Flow cytometry data used for ADCC. [file crc-22-0356-s02.pdf]

A

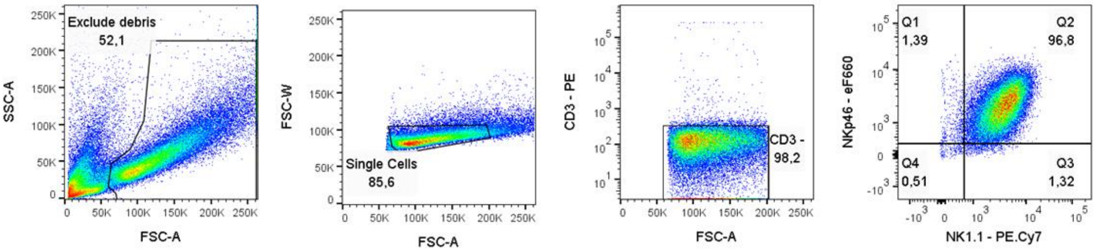

Supplementary figure 2. Flow cytometry data used for ADCC. (A) Characterization of NK population at day 5 derived from ex vivo material. Gating was done on unstained splenocytes at day 5 and its respective fluorescence-minus-one sample. First, NK cells were gated based on FSC-A / SSC-A properties. Next, single cells were gated based FSC-A / FSC-W. NK population were gated as CD3 negative. Next to the $CD3^-$ population, NK population are found at $NKp46^+$ $NK1.1^+$ gate.
