# Supplementary material for: Mouse IgG2a Isotype Therapeutic Antibodies Elicit Superior Tumor Growth Control Compared with mIgG1 or mIgE
Source: Cancer Res Commun. 2023 Jan 23;3(1):109–18. doi: 10.1158/2767-9764.CRC-22-0356 (PMC10035513; doi:10.1158/2767-9764.CRC-22-0356)
Supplement: Supplementary Figure SF3 — Confirmation of OT-1 cells activation by flow cytometry. [file crc-22-0356-s03.pdf]

A

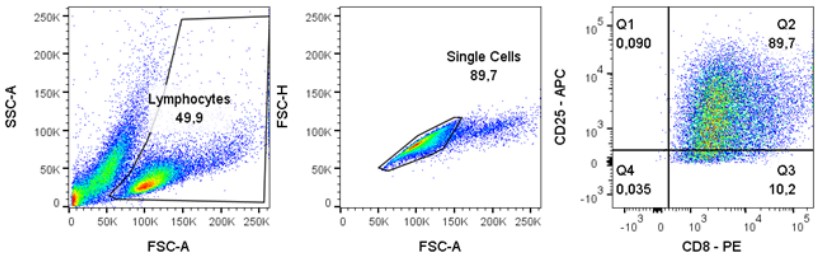

Supplementary figure 3. Confirmation of OT-1 cells activation by flow cytometry. (A) Characterization of OT-1 cells at day 3 before injection. Gating was done based on FSC-A / SSC-A properties. Next, single cells were gated based FSC-A / FSC-H. OT-1 cells were gated as CD8 positive and activated cells as CD25 positive. CD8 and CD25 quadrants based on non-stained sample.
