# Supplementary material for: Mouse IgG2a Isotype Therapeutic Antibodies Elicit Superior Tumor Growth Control Compared with mIgG1 or mIgE
Source: Cancer Res Commun. 2023 Jan 23;3(1):109–18. doi: 10.1158/2767-9764.CRC-22-0356 (PMC10035513; doi:10.1158/2767-9764.CRC-22-0356)
Supplement: Supplementary Figure SF4 — No synergistic effected is achieved combining anti-thy1.1 antibodies with adoptive cell transfer of activated OT-1s. [file crc-22-0356-s04.pdf]

A

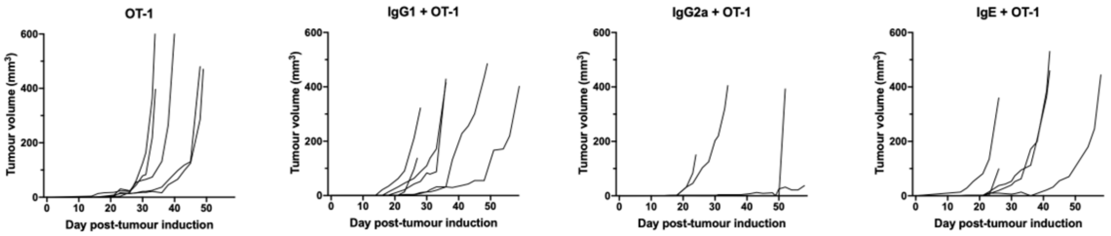

Supplementary figure 4. No synergistic effected is achieved combining anti-thy1.1 antibodies with adoptive cell transfer of activated OT-1s. C57BL/6 mice were subcutaneously injected with 50 000 B16-OVA-Thy1.1 cells in the flank and were treated with anti-Thy1.1 IgG1, IgG2a or IgE antibodies. Mice received the combination treatment consisting of anti-Thy1.1 antibodies and adoptive cell transfer of activated OT-1 cells. (A) Tumor growth curves. n=5-6, the experiment was done once.
