# Supplementary material for: Mouse IgG2a Isotype Therapeutic Antibodies Elicit Superior Tumor Growth Control Compared with mIgG1 or mIgE
Source: Cancer Res Commun. 2023 Jan 23;3(1):109–18. doi: 10.1158/2767-9764.CRC-22-0356 (PMC10035513; doi:10.1158/2767-9764.CRC-22-0356)
Supplement: Supplementary Table ST1 — Amino acid sequences of anti-Thy1.1 antibodies. [file crc-22-0356-s05.pdf]

Supplementary Table 1. Amino acid sequences of anti-Thy1.1 antibodies. Signal peptides are highlighted in grey, and LALA-PG mutations in cyan.

**Anti-Thy1.1 IgG2a HC**
MAVLGLLFCLVTFPSCVLSEIQLQQSGPELMKPGASVKISCKASGYSFTSYYMDWVKQSHGKNLEWIGYIDPFN<br>GDTSYNQKFKDKATLTVDKSSSTAYMHLSSLTSEDSAVYYCARGIYYGYGGYFDYWGQGTTLTVSSAKTTAPSVY<br>PLAPVCGDTTGSSVTLGCLVKGYFPEPVTLTWNSGSLSSGVHTFPAVLQSDLYTLSSSVTVTSSTWPSQSITCNVA<br>HPASSTKVDKKIEPRGPTIKPCPPCKCPAPNLLGGPSVFIFPPKIKDVLMISLSPIVTCVVVDVSEDDPDVQISWFV<br>NNVEVHTAQTQTHREDYNSTLRVVSALPIQHQDWMSGKEFKCKVNNKDLPAPIERTISKPKGSVRAPQVYVLP<br>PPEEEMTKKQVTLTCMVTDFMPEDIYVEWTNNGKTELNYKNTEPVLDSDGSYFMYSKLRVEKKNWVERNSYS<br>CSVVHEGLHNHHTTKSFSRTPGK
**Anti-Thy1.1 IgG2a LALA-PG HC**
MAVLGLLFCLVTFPSCVLSEIQLQQSGPELMKPGASVKISCKASGYSFTSYYMDWVKQSHGKNLEWIGYIDPFN<br>GDTSYNQKFKDKATLTVDKSSSTAYMHLSSLTSEDSAVYYCARGIYYGYGGYFDYWGQGTTLTVSSAKTTAPSVY<br>PLAPVCGDTTGSSVTLGCLVKGYFPEPVTLTWNSGSLSSGVHTFPAVLQSDLYTLSSSVTVTSSTWPSQSITCNVA<br>HPASSTKVDKKIEPRGPTIKPCPPCKCPAPNAAGGPSVFIFPPKIKDVLMISLSPIVTCVVVDVSEDDPDVQISWFV<br>NNVEVHTAQTQTHREDYNSTLRVVSALPIQHQDWMSGKEFKCKVNNKDLGAPIERTISKPKGSVRAPQVYVLP<br>PPEEEMTKKQVTLTCMVTDFMPEDIYVEWTNNGKTELNYKNTEPVLDSDGSYFMYSKLRVEKKNWVERNSYS<br>CSVVHEGLHNHHTTKSFSRTPGK
**Anti-Thy1.1 IgG1 HC**
MAVLGLLFCLVTFPSCVLSEIQLQQSGPELMKPGASVKISCKASGYSFTSYYMDWVKQSHGKNLEWIGYIDPFN<br>GDTSYNQKFKDKATLTVDKSSSTAYMHLSSLTSEDSAVYYCARGIYYGYGGYFDYWGQGTTLTVSSAKTTPPSVY<br>PLAPGSAAQTNSMVTLGCLVKGYFPEPVTVTWNSGSLSSGVHTFPAVLQSDLYTLSSSVTVPSSTWPSETVTCN<br>VAHPASSTKVDKKIVPRDCGCKPCICTVPEVSSVFIFPPKPKDVLTITLTPKVTCVVVDISKDDPEVQFSWFVDDVE<br>VHTAQTQPREEQFNSTFRSVSELPIMHQDWLNGKEFKCRVNSAAFPAPIEKTISKTKGRPKAPQVYTIPPPKEQ<br>MAKDKVSLTCMITDFFPEDITVEWQWNGQPAENYKNTQPIMDTDGSYFVYSKLNVQKSNWEAGNTFTCSVL<br>HEGLHNHHTEKSLSHSPGK
**Anti-Thy1.1 IgE HC**
MAVLGLLFCLVTFPSCVLSEIQLQQSGPELMKPGASVKISCKASGYSFTSYYMDWVKQSHGKNLEWIGYIDPFN<br>GDTSYNQKFKDKATLTVDKSSSTAYMHLSSLTSEDSAVYYCARGIYYGYGGYFDYWGQGTTLTVSSASIRNPQLY<br>PLKPCKGTASMTLGCLVKDYFPNPVTVTWYSDSLNMSTVNFPALGSELKVTTSQVTSWGKSAKNFTCHVTHPP<br>SFNESRTILVRPVNITEPTLELLHSSCDPNAFHSTIQLYCFIYGHILNDVSVSWLMDDREITDTLAQTVLIKEEGKLA<br>STCSKLNITEQQWMSESTFTCKVTSQGVDYLAHTRRCPDHEPRGVITYLIPPSPLDLYQNGAPKLTCLVVDLESEK<br>NVNVTWNQEKKTSVSASQWYTKHHNNATTSITSILPVVAKDWIEGYGYQCIVDHPDFPKPIVRSITKTPGQRSA<br>PEVYVFPPPEEESEDKRTLTCLIQNFFPEDISVQWLGDGKLISNSQHSTTTPLKSNGSNQGFFIFSRLEVAKTLWT<br>QRKQFTCQVIHEALQKPRKLEKTISTSLGNTSLRPS
**Anti-Thy1.1 LC**
MSVLTQVLALLLLWLTGARCDIVLTQSPASLAVSLGQRATISCRASDSVDSFGNSFMHWFQQKPGQPPKLLIYR<br>ASTPESGIPARFSGSGSRTDFTLTISPVEADDVATYYCQQSIEDPFTFGGGTKLEIKRADAAPTVSIFPPSSEQLTSG<br>GASVVCFLNNFYPKDINVKWKIDGSERQNGVLNSWTDQDSKDSTYSMSSTLTLTKDEYERHNSYTCEATHKTST<br>SPIVKSFNRNEC
