# Supplementary material for: Mouse IgG2a Isotype Therapeutic Antibodies Elicit Superior Tumor Growth Control Compared with mIgG1 or mIgE
Source: Cancer Res Commun. 2023 Jan 23;3(1):109–18. doi: 10.1158/2767-9764.CRC-22-0356 (PMC10035513; doi:10.1158/2767-9764.CRC-22-0356)
Supplement: Supplementary Table ST2 — Amino acid sequence of designed Thy1.1-MHC-1 construct [file crc-22-0356-s06.pdf]

Supplementary Table 2. Amino acid sequence of designed Thy1.1-MHC-1 construct

**Thy1.1-MHC-1**
MNPVISITLLLSVLQMSRGQRVISLTACLVNQNLRLDCRHENNTNLPIQHEFSLTREKKKHVLSGTLGVPEHTYRSRVNLFSDRFIKVLTLANFTTKDEGDYMCELRVSGQNPTSSNKTINVIRDKLVKCGKEEPPSSTKTNTVIIAVPVVLGAVVILGAVMAFVMKRRRNTGGKGGDYALAPGSQSSDMSLPDCKV

Legend: Signal peptide – Thy1.1 without its propeptide – connecting peptide – transmembrane domain of MHC-1 – cytoplasmic domain of MHC-1
